# A human cell-based SARS-CoV-2 vaccine elicits potent neutralizing antibody responses and protects mice from SARS-CoV-2 challenge

**DOI:** 10.1080/22221751.2021.1957400

**Published:** 2021-08-12

**Authors:** Xiangchuan He, Longfei Ding, Kangli Cao, Haoran Peng, Chenjian Gu, Yutang Li, Duoduo Li, Lanlan Dong, Xiujing Hong, Xiangwei Wang, Meilan Fu, Chenli Qiu, Cuisong Zhu, Ziling Zhang, Shu Song, Chenguang Wang, Zhengfan Jiang, Youhua Xie, Zhongtian Qi, Chen Zhao, Ping Zhao, Xiaoyan Zhang, Jianqing Xu

**Affiliations:** a Shanghai Public Health Clinical Center & Institutes of Biomedical Sciences, Fudan University, Shanghai, People’s Republic of China; b Department of Microbiology, Second Military Medical University, Shanghai, People’s Republic of China; c Department of Medical Microbiology and Parasitology, School of Basic Medical Sciences, Shanghai Medical College, Fudan University, Shanghai, People’s Republic of China; d Key Laboratory of Cell Proliferation and Differentiation of the Ministry of Education, School of Life Sciences & Peking-Tsinghua Center for Life Sciences, Peking University, Beijing, People’s Republic of China

**Keywords:** SARS-CoV-2, cell-based vaccines, K562-S, mouse model, non-human primate model, neutralizing antibody, protection

## Abstract

To curb the pandemic of coronavirus disease 2019 (COVID-19) caused by the severe acute respiratory syndrome coronavirus 2 (SARS-CoV-2), multiple platforms have been employed toward a safe and highly effective vaccine. Here, we develop a novel cell-based vaccine candidate, namely K562-S, by utilizing human cell K562 as a cellular carrier to display Spike (S) protein of SARS-CoV-2 on the membrane. Analogous to the traditional inactivated vaccine, K562-S cells can be propagated to a large scale by culturing and completely lose their viability after exposure to X-ray irradiation or formalin. We in turn demonstrated high immunogenicity of formalin-inactivated K562-S vaccine in both mouse and non-human primates and its protective efficacy in mice. In mice, immunization with inactivated K562-S vaccines can elicit potent neutralizing antibody (nAb) responses persisting longer than 5 months. We consequently showed in a hACE2 mouse model of SARS-CoV-2 infection that a two-shot vaccination with adjuvanted K562-S rendered greater than 3 log reduction in viral lung load and concomitant ameliorated lung pathology. Of importance, the administration of the same regimen in non-human primates was able to induce a neutralizing antibody titer averaging three-fold higher relative to human convalescent serum. These results together support the promise of K562-based, S-protein-expressing vaccines as a novel vaccination approach against SARS-CoV-2. Importantly, with a powerful capacity to carry external genes for cell-based vectors, this platform could rapidly generate two- and multiple-valent vaccines by incorporating SARS-CoV-2 mutants, SARS-CoV, or MERS-CoV.

## Introduction

SARS-CoV-2 (severe acute respiratory syndrome coronavirus-2), also known as 2019-nCoV, has been identified as the causative agent for the Coronavirus Disease 2019 (COVID-19) [1]. SARS-CoV-2 belongs to the betacoronavirus genus of the coronaviridae family, closely related to two highly pathogenic viruses, SARS-CoV and middle east respiratory syndrome coronavirus (MERS-CoV), which account for the previous outbreak in 2003 and 2012, respectively [2]. Coronaviruses are enveloped viruses featuring a large, positive-sense single-stranded RNA genome that encodes four major structural proteins, including the spike (S) glycoprotein protruding from the surface of the virus.

The S-protein contains two subunits with distinct functions: S1 subunit is responsible for cell entry mainly through recognizing and binding to the human angiotensin enzyme 2 (hACE2) by the receptor-binding domain (RBD), and S2 subunit acts subsequently to mediate membrane fusion [3,4]. The isolation of a handful of human monoclonal antibodies with potent SARS-CoV-2 neutralizing activity from human convalescent serum (HCS) substantiated the feasibility of vaccination and highlighted S-protein as the desirable viral target of vaccination, with epitope mapping showing the majority of the neutralizing epitopes lying in S-protein, particularly in its RBD [5–10]. Consequently, most of the SARS-CoV-2 vaccine candidates used the S-protein or RBD as the immunogen, aiming to induce robust neutralizing antibody responses [4,11,12].

As the oldest member of vaccine family with a history of success, inactivated vaccines remain a major type of vaccines employed against many pathogens including influenza and SARS-CoV-2. For making inactivated vaccines, virus or bacteria was propagated in culture and then exposed to chemical or physical agents to destroy its replicative activity while maintaining a normal display of its surface proteins and thus leave the immunogenicity largely intact [13]. Inspired by the working mechanism of inactivated vaccine, we herein added to the roadmaps of SARS-CoV-2 vaccine a new avenue, that is, using cultured human cells as a carrier to express S-protein in its native membrane-bound form with their safety as vaccines being secured by inactivation without loss of immunogenicity. For this exploration, we chose the human erythroleukemic K562 cell line as the S-protein cellular carrier because of its two distinct features. First, it lacked expression of HLA moieties, rendering it an exquisite target for the NK cells [14]. We envisioned that NK cell-induced K562 death might engage a cascade of immune responses, including cytokine release and recruitment/maturation of antigen-presenting cells for the presentation of K562-encoded proteins, thus eliciting protective adaptive immunity provided by B cells and T cells. Secondly, K562 cells do not express RBC A/B blood type antigen, allowing usage for all blood types [15]. Noteworthy, during our experimental examination of S-expressing K562 as a human cellular vaccine against SARS-CoV-2, the same strategy was also conceptually proposed by Ji et al. who used “I” cells-immunogen-carrying cells- to relate the underlying mechanism [16]. Of importance, irradiation-mediated inactivation has been demonstrated to be sufficient for securing the biosafety of transfusion of K562 cells. Indeed, irradiated K562 cells expressing granulocyte-macrophage colony-stimulating factor (GM-CSF) have been clinically assessed for treating myelodysplastic syndromes (MDS) and chronic myeloid leukaemia (CML) patients, with noted haematologic improvements and no serious adverse events [17–19]. Accordingly, we engineered K562-S to stably express S-protein, namely K562-S, and subsequently study its efficacy after inactivation, either alone or in combination with different adjuvants, as a vaccine against SARS-CoV-2 in pre-clinical animal models. Our data present strong evidence supporting the promise of K562-S as a new effective vaccine for the prevention of COVID-19 and warranted its future clinical development.

## Materials and methods

### Study design and animals

The primary objective of this study was to characterize the immunogenicity and efficacy of K562-S, an inactivated whole-cell vaccine derived from human K562 cells to express membrane S-protein of SARS-CoV-2, with various adjuvants and modalities in mice and rhesus macaques. The adjuvants being tested included Alum, MnJ, CpG, Alum or MnJ plus CpG. The attempted two-shot modalities included adjuvanted, inactivated K562-S as both prime and boost, or the prime being replaced with either S-expressing DNA vector. Animals were randomly assigned to experimental and control groups, with the size of groups in the rhesus macaque study being limited by the availability of animals. End points were pre-specified before the start of each study and in some cases were selected based on the primary objective of characterizing immune responses elicited by K562-S vaccination.

### Cell lines and viruses

Human chronic myelogenous leukaemia K562 cell line (ATCC, CCL-243), human embryonic kidney HEK293 T cell line (ATCC CRL-3216) and African green monkey kidney epithelial Vero E6 cell line (ATCC CRL-1586) were purchased from American Type Culture Collection (ATCC). K562 cells were maintained in R10 medium-RPMI 1640 (Corning,10–040-CVR) supplemented with 10% fetal bovine serum (FBS) (BI, 04–001–1acs) and 1% penicillin–streptomycin (PS) (Corning, 30–002-CI), whereas D10 medium-FBS and PS supplemented DMEM (Corning, 10–013-CV) was used for culturing HEK293 T and Vero E6 cells. The generation of SARS-CoV-2 pseudovirus carrying a firefly luciferase reporter gene was described in our previous publication [5,20]. SARS-CoV-2 strain CHN/Shanghai_CH-02/2020 (GenBank: MT627325.1) and SARS-CoV-2-SH01, both propagated and titrated in Vero E6 cells, were respectively used for mouse challenge study and authentic virus neutralizing assay.

### Confirmation of key characteristics of K562 cells

K562 cells were characterized for three key features previously reported, namely lacking expression of HLA molecules, expression of CD71 surface marker, and free of human red blood cell (RBC) A and B antigens. For HLA detection, K562 cells were subjected to either staining of HLA-DR APC (eBioscience, E021498) with purified B cells as positive control or staining of HLA-A/B/C APC (Biolegend, W6/32) with 293 T as positive control; for CD71 detection, cells were stained with CD71 PE/Cy7 (Biolegend, 334112). The stained cells were subsequently used for flow cytometry analysis. The expression of RBC antigens was measured by immunoblotting of lysates derived from K562 cells versus those from human RBC using anti-blood group AB antigen antibody (Santa Cruz, sc-52370).

### Construction, verification, and characterization of K562-S vaccine

Codon-optimized DNA sequences encoding SARS-CoV-2 spike (S) protein (YP_009724390.1) were synthesized (Generay Biotech Co., Ltd), and cloned into pHAGE-puro lentiviral vector (Addgene, 118692) to generate pHAGE-S-puro plasmid. Subsequently, 3 μg of pHAGE-S-puro plasmid, combined with 3 μg of the VSV-G envelope plasmid PMD2.G (Addgene, 12259) and 9 μg of the packaging plasmid psPAX2 encoding gag-pol (Addgene,12260), were used to transfect 10 cm of HEK293 T cell culture (6 × 10^6^ cells) by TurboFect transfection reagent (Thermo Scientific, R0531). The lentivirus-containing supernatant was harvested 48 h after transfection, filtered through a 0.45-μm filter (PALL,4614), and concentrated by Lenti-X concentrator (TaKaRa,631232). Polybrene-mediated transduction of K562 cells with the resultant lentivirus stock was performed in 12-well format by centrifugation at 1000 g for 2 h at 32°C. After overnight incubation at 37°C, the culture medium was replaced with a fresh R10 medium and cells were cultured for additional 48 h before switching to R10 containing 2 μg/ml puromycin for selection. The cells that remained after puromycin selection were subjected to single-cell sorting by flow cytometry based on recognition by human ACE2 protein. In brief, 1 × 10^6^ of K562-S cells were incubated with biotin-labelled ACE2 protein (Novoprotein, CY51; final concentration of 20 µg/ml) in 100 µl for 30 min at room temperature (RT), washed twice in PBS before addition of PE-labelled streptavidin-antibody to a final concentration of 4 µg/ml (Biolegend, 405203) for 30-min incubation. The stained cells were then washed twice in PBS before sorted by a FACSAriaIII cytometer (BD Biosciences, USA) for sorting.

After expansion, individual K562-S clones were characterized for S-protein expression. The expression of S-protein was determined on 1 × 10^6^ K562-S cells by western blotting analysis using a rabbit anti-S antibody (Sino Biological, 40591-T62), with purified recombinant SARS-CoV-2 S (ECD) (Genscript, Z03481) as the reference protein. The amount of S-protein in K562-S lysate was further quantitated by a SARS-CoV-2 S-protein ELISA detection kit (Biodragon, BF03087). The homogenicity for S-protein expression were examined by flow cytometry following hACE2 staining. One clone with nearly 100% of cells analyzed being positive for ACE2 staining was selected as seed for preparation of K562-S vaccines through continuous propagation.

The selected K562-S clone was characterized for its membrane presentation of S-protein by immunofluorescence. In brief, ImmEdge^TM^ hydrophobic pen was used to encircle an area of 1 cm ×  2 cm on a positively charged adhesion slide, to which 100 μl of K562-S cell suspension (2 × 10^5^ cells) was even added followed by air drying and were subsequently submitted to staining with a mouse anti-S2 (SARS-CoV-2) primary antibody (1:1000, GeneTex, GTX632604) and a goat anti-mouse IgG secondary antibody labelled with Alexa Fluor 594 (1:2000, Invitrogen, A11032). The stained cells were mounted with ProLong™ Gold Antifade Mountant with DAPI (Invitrogen, P36931), covered with a coverslip, and imaged using a TissueFAXS 200 flow-type tissue cytometer (TissueGnostics GmbH, Vienna, Austria).

### Inactivation of K562-S cells for vaccine preparation

The inactivation of K562-S cells was achieved either by formalin fixation or X-ray irradiation. For formalin-mediated inactivation, cells were counted and, following collection by centrifugation, resuspended in 1% formalin to a final concentration of 1 × 10^7^ cells/ml. After fixation for 10 min at RT, the cells were washed 4 times with PBS to remove the residual formalin. For X-ray irradiation-mediated inactivation, the same counting and centrifugation steps were used, but cell resuspension was made in PBS and subsequently subjected to X-ray irradiation with doses of 50Gy by the use of an X-ray irradiator (RAD SOURSE RS2000 PRO). Where indicated, the inactivated K562-S cells were stored in −70°C or RT. Live K562-S control was freshly prepared by resuspending K562-S cells in PBS after centrifugation and PBS washing.

### Animal experiments

All animal experiments in this study were approved by the Institutional Animal Care and Use Committee (IACUC) of Shanghai Public Health Clinical Center and Second Military Medical University (SMMU). Pathogen-free six-week-old female C57/BL6 and ICR mice, purchase from Shanghai Jihui biological Co., Ltd, were housed in the animal facility of Shanghai Public Health Clinical Center (SPHCC). SARS-CoV-2 challenge studies were performed on C57/BL6 mice carrying a transgene (CAG-human ACE2-IRES-Luciferase), abbreviated as hACE2 mouse throughout the manuscript, in which both human ACE2 gene and luciferase reporter gene is driven by CAG promoter while the latter is independently translated via an IRES. The six-week-old aged hACE-2 mice were maintained at BSL-3 laboratory in the Second Military Medical University (SMMU). Three/four-year-old female rhesus macaques, purchased from a domestic source (Ningbo, China), were housed in the animal facility of SPHCC. Mouse studies involving SARS-CoV-2 infection were conducted in the BSL-3 facilities in SMMU.

### Immunization and challenge of mice

Female C57BL/6 or ICR mice (6–12 weeks old) were submitted to indicated immunization regimen consisting of various forms of K562-S vaccines (live, inactivated, inactivated and adjuvanted) and/or S-encoding DNA vaccine derived from psv1.0 plasmid (DNA-S), which was constructed in our lab by routine cloning. A single dose of K562-S contained 1 × 10^6^ K562-S cells and the following adjuvant, individually or in combination, where indicated: 500 μg of aluminium hydroxide gel adjuvant (Alum) (InvivoGen,vac-alu-250), 100 μg of Mn Jelly (MnJ) (MnStarter Biotechnology Co., Ltd.), or 30 μg of CpG ODN 1826 (CpG) (InvivoGen,vac-1826-1). A single dose of DNA-S contained 10 μg DNA without adjuvant. Both K562-S and DNA-S vaccines were administered via intramuscular injection in 100 μl volume per dose. The sera were collected at indicated time points for characterization of antibody response. In the experiments where T cell responses were assessed, the spleens were collected and teased apart to prepare a single-cell suspension of splenocytes for peptide stimulation and subsequent ELISpot analysis.

The SARS-CoV-2 challenge experiment was performed on female hACE2-C57BL/6 mice that received a homologous prime and boost at 3-week intervals consisting of either sham K562-WT or FI-inactivated K562-S mixed with Alum, Alum plus CpG, or MnJ plus CpG. Each experiment group contained six animals. At 4 weeks after the priming immunization, the animals were intranasally inoculated with 1 × 10^4^ PFU of SARS-CoV-2 (CHN/Shanghai_CH-02/2020, GenBank:MT627325.1); virus inoculations were performed under anaesthesia induced and maintained with ketamine hydrochloride and xylazine to minimize animal suffering. Lung pathology and lung viral loads were determined at day 3 post challenge.

### Immunization of rhesus macaques

Two prime-boost immunizations with 4-week interval regimens were tested in rhesus macaques, one with DNA-S as prime and K562-S adjuvanted with MnJ plus CpG as boost (*n* = 3) and the other with K562-S adjuvanted with MnJ plus CpG as both prime and boost. A single dose of K562-S and DNA in 400 μl volume contained 1 × 10^7^ cells and 1 mg DNA, respectively; the amount of adjuvant per dose was 1 mg for MnJ and 300 μg for CpG ODN 2006 (InvivoGen, #tlrl-2006-5) adjuvants. Both priming and booster vaccines were administered by intramuscular injection into the quadriceps muscles of hind limbs. Serum was collected just before immunization and at study week 2, 4, 5, 6 and 8 for characterization of antibody response. Animals were carefully monitored for general health, including activity and appetite, and for evidence of reactogenicity-swelling and redness-at the vaccine injection site. None of the animals became severely ill during the course of the study, and none required euthanasia.

### Enzyme-linked immunosorbent assay (ELISA)

An ELISA was used to measure the RBD binding antibody titers of serum samples. Briefly, 96-well ELISA plates were coated with 0.5 mg/ml of recombinant SARS-CoV-2 RBD protein (Genscript Z03483-1) at 4°C overnight. Plates were washed with PBS containing 0.5% Tween-20 (PBS-T) and blocked with 5% non-fat milk in PBST (5% milk/PBST) for 2 h at RT. Mouse, rhesus macaque, or human serum samples were serially diluted from 1/100 in 2-fold dilutions in 5% milk/PBST and were added to each well in triplicate before incubation at RT for 3 h. Following incubation, the plates were extensively washed with PBST. For assessment of total IgG, HRP-conjugated goat anti-mouse IgG (Zsbio, ZB-5305, 1:5000), goat anti-monkey IgG (Biodragon, BF03037,1:3000), or goat anti-human IgG (Zsbio, ZB-2305, 1:5000) was added at a dilution of 1:5000 in 5% milk/PBST and incubated at RT for l h. After extensive washing plates with PBST, the substrate OPD, prepared by dissolving a SIGMAFAST OPD tablet (Sigma, SLCC0308) in 20 ml deionized water, was added to the plates and incubated at RT for 5 mins. The reactions were terminated by the addition of 1M H2SO4 to terminate the reaction and then read at OD490 with a Synergy Microplate Reader (Bio-Tek, Winooski, VT). For the detection of IgG1 and IgG2 in the mouse serum sample, due to lack of suitable HRP-conjugated anti-mouse IgG1 and IgG2 antibodies, we first incubated the plates with rat anti-mouse IgG1, or IgG2 (Biolegend, 406603 & 407103, 1:2500 dilution in 5% milk/PBST) at RT for 2 h. After removal of the antibodies and extensive washing with PBST, HRP-conjugated anti-rat IgG (Zsbio, ZB-2307) was added at a dilution of 1:5000 in 5% non-fat milk- PBST and incubated at RT for 1 h, followed by the final step of colour development as described above. ELISA endpoint titers were defined as the highest reciprocal serum dilution that yielded an absorbance 2-fold greater than background values. Data were analyzed using GraphPad Prism 8.0.

### IFN-gamma ELISpot assay

The T cell responses of vaccinated mice were analyzed using a mouse IFN-gamma ELISPOT assay set (BD Bioscience, #551083) following the manufacture’s protocol. In brief, 96-well ELISpot plates were pre-coated with 5 μg/mL of anti-mouse IFN-γ antibody overnight at 4°C. After rinsing with R10 culture medium, plates were blocked with R10 at RT for 2 h. Single-cell suspensions of splenocytes in R10 were then added to the plate (2 × 10^5^ cells/well) and stimulated with RBD-derived peptide pool(s)-15-mer peptides overlapped by 11 amino acids covering the entire RBD sequence (5 μg/well). Each assay was performed in duplicate. Peptide stimulation lasted 18–24 h with plates being placed in a humidified 5% CO2 incubator at 37°C. Following the incubation, the plates were sequentially washed twice with pre-cooled water and three times with PBST before the addition of biotinylated anti-mouse IFN-γ antibody (2 μg/ml) for 2 h of incubation. Streptavidin conjugated horseradish peroxidase was then added at 1:100 dilution and, after 1 h incubation at RT, the plates were washed with PBS and subjected to spot development with AEC substrate reagent (BD Bioscience, #551951). The reaction was stopped by rinsing the plates with water before allowing the plate to dry 24 h in darkness. Wells were imaged and spot-forming cells (SFCs) were determined with a Biospot plate reader (ChampSpot III, Beijing SageCreation Science Co., Ltd).

### Human convalescent serum

Convalescent serum samples (*n* = 7; numbered 947, 950, 952, 953, 964, 966, 967) from patients who had recovered from SARS-CoV-2 infection were obtained from Shanghai Public Health Clinical Center and stored at −80°C until analysis. The information of patient details was provided in Supplementary Table 1. Sera were analyzed for titers of ant-RBD binding antibodies and neutralizing activities against pseudovirus and authentic SARS-CoV-2 virus.

### Pseudovirus neutralization assay

The reagents and procedures for generation of SARS-CoV-2 pseudovirus bearing full length of SARS-CoV-2 spike protein (YP_009724390.1) and a luciferase reporter gene were described in our previous publication [20]. In brief, HEK293 T cells were co-transfected with pNL4-3.Luc.R-E-(NIH AIDS Reagent Program, cat#3418) and pcDNA3/SARS-CoV2-S using TurboFect reagent (Thermo Scientific, cat#R0531). Twelve hours post-transfection, culture medium was replaced with fresh D10 medium and incubated for additional 48 h. The pseudovirus-containing supernatant was harvested and subjected to centrifugation. The cleared supernatants were filtered through 0.45 μm, aliquoted, and stored at −80°C until use. An aliquot of supernatant was submitted to titration assay to determine the dilution needed in the neutralization assay. Neutralization assays were subsequently performed in triplicate for assessment of neutralizing antibody against SARS-CoV-2. Specifically, serum was serially diluted in D10 medium after heat inactivation and then mixed with equal volume (50 μl) of diluted pseudovirus. After incubation at 37°C for 1 h, the serum-pseudovirus mixtures were transferred to wells of 96-well plate seeded 12 h earlier with 2 × 10^4^ hACE2-293 T cells. Forty-eight hours later, cells were assayed using Bright-Glo™ Luciferase Assay System (Promega), with the relative light units (RLUs) being read on a luminometer (Promega GloMax 96). The neutralization antibody titers were calculated as 50% inhibitory doses (ID50), expressed as the highest dilution of sample which resulted in a 50% reduction in RLUs relative to virus control wells (virus control abbreviated as VC, RLUs of no sera samples wells) after subtraction of background (cell control abbreviated as CC, RLUs of no virus wells).

### Authentic neutralization assay

All experiments involving authentic SARS-CoV-2 virus were performed in a BSL-3 laboratory. Serial dilutions of heat-inactivated serum samples were incubated with 200 PFU of SARS-CoV-2 SH01 virus at 37°C for 1 h. The mixture was then transferred to wells of 96-well plates in triplicate that was seeded with 4 × 10^4^ Vero E6 cells 12 h earlier, and the plate was then incubated for 2 days at 37°C in a humidified 5% CO2 incubator. After the incubation, the cells were inspected under inverted light microscopy for cytopathic effect (CPE) to assess the inhibitory activity of each dilution. Reported were 50% inhibitory doses (ID50), expressed as the highest dilutions of sera capable of completely preventing virus-induced CPE in at least 50% of the wells.

### Quantitative reverse transcription polymerase chain reaction (qRT-PCR)

Viral loads in lungs of infected mice were determined by quantitative real-time PCR (qRT-PCR). In brief, total RNA was extracted from pulverized lung tissues of infected mice using Trizol (Thermo) in combination with Direct-zol RNA Miniprep Plus kit (Zymo Research) and then used to synthesize cDNA with Maxima Reverse Transcriptase using random hexamers (Thermo). The resultant cDNA was subsequently proceeded to SYBR green-based RT–PCR analysis for SARS-CoV-2 N transcript using the GoTaq qPCR Master Mix (Promega, Charbonnieres, France) on a Bioer real-time PCR system. The N-specific primers were: forward, 5′-GGG GAA CTT CTC CTG CTA GAA T-3′; reverse, 5′-CAG ACA TTT TGC TCT CAA GCT G-3′. The conditions for PCR amplification were as follows: 42°C for 5 min; 95°C for 10 s; 40 rounds of 95°C for 10 s and 60°C for 30 s. The N transcripts were quantified based on a standard curve generated from measurements of serial dilutions from a reference plasmid containing the full-length cDNA of the SARS-CoV-2 N gene. The assay sensitivity was determined to be 23 plasmid copies, equivalent to a cycle threshold (CT) of 35.

### Histopathology

The hACE2 transgenic mice were euthanized 3 days following SARS-CoV-2 challenge. The lungs were fixed in 4% formalin and subsequently embedded in paraffin, followed by sectioning to 5 μm with a microtome. Sections were stained with haematoxylin and eosin (H&E) and scanned using TissueFAXS Confocal Plus 200 (Tissue Gnostics). The acquired images were analyzed by Strata Quest 6.0X software to assess individual characteristics of lung pathology, and pathological scores were subsequently calculated following the method detailedly described in our previous publication [21].

### Statistical analyses

Statistical analyses were performed with GraphPad Prism software (Prism V8.0, GraphPad). Two-tailed Student’s t-test or nonparametric Mann–Whitney test was used to compare differences between two groups, and Kruskal–Wallis test with Dunn’s adjustment was applied when comparing more than two groups. Correlation analysis was conducted with Spearman’s test with the correlation coefficient *r*, ranging from −1 to +1, measuring the strength and direction of a linear relationship between two variables. Kaplan-Meier survival curves were compared by using log-rank test. The number of biological replicates was indicated in both text and figure legends. A *P* value of ≤0.05 was considered statistically significant.

## Results

### Design and construction of human K562 cell-based vaccine

**Figure 1.**
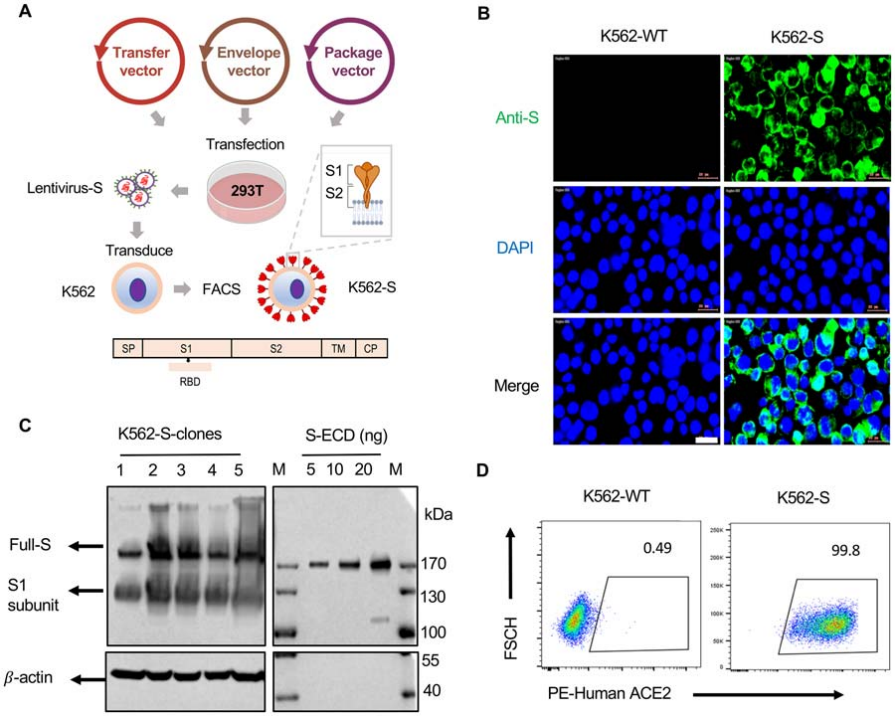
Design and construction of the K562 human cell-based vaccine. (A) Schematic depiction of the construction of K562-S vaccine, a whole human cell-based vaccine expressing the full-length spike (S) protein of SARS-CoV-2 as antigen. Verification of universal expression of S-protein and its membrane presentation of the selected K562-S clones by immunofluorescence analysis (B), western blotting (C) and flow cytometry (D). For (B), cells were fixed and immunostained for S-protein (Green) following procedures described under “Materials and methods”. For (C), the lysates derived from 1 × 10^6^ K562-S cells, together with the indicated amounts of purified extra cellular domain of spike protein (ECD) S-protein, were subjected to denaturing gel electrophoresis, followed by immunoblotting with a rabbit anti-S antibody. For (D), K562-S cells and original K562 (K562-WT) cells were stained with PE-labelled purified human ACE2 protein, followed by flow cytometry.

To generate a human cell-based SARS-CoV-2 vaccine, K562 cells were infected by a lentivirus encoding a codon-optimized sequence for full-length SARS-CoV-2 Spike (S) protein with intact signal peptide (Figure 1(A)). The expression of S-protein on the surface of infected cells was successfully detected by immunofluorescence staining (Figure 1(B)), and further validated by western blotting analysis of K562 cells, wherein both full length of S-protein and a truncated form likely representing the S1 subunit generated after furin-mediated cleavage were readily detected (Figure 1(C)). We further characterized cloned K562 cells by staining with biotin-labelled purified human ACE2 protein followed by flow cytometry analysis. Nearly, all the cells were stained, verifying that S-protein expressed on the K562 surface retains the native property and also demonstrating that S-protein expression is constantly maintained after extensive cell expansion (Figure 1(D)). We also confirmed by western blotting analysis that MHC molecules (HLA-DR; HLA-A/B/C) and RBC antigens are absent in K562 cells (Figure S1 A,B).

### Inactivation methods for production of K562-S vaccines with maintained immunogenicity

**Figure 2.**
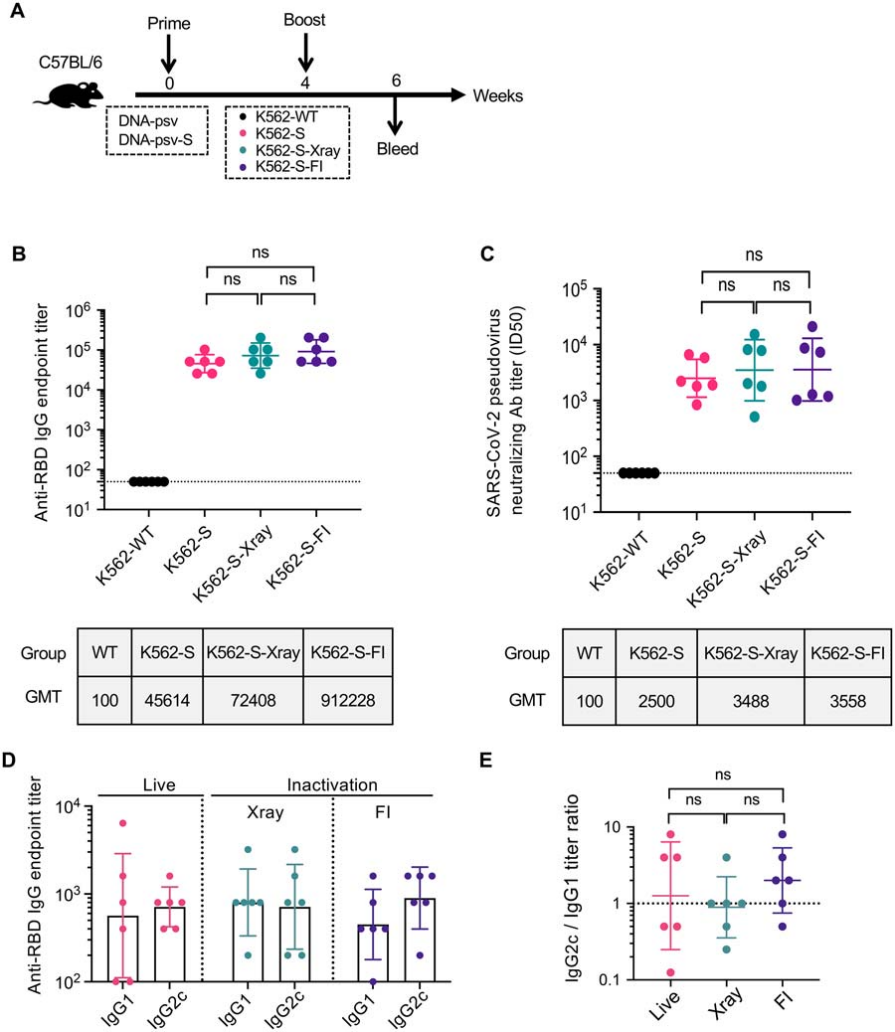
The effect of inactivation on the immunogenicity of K562-S vaccines. (A) Experimental scheme. Female C57/BL/6 (*n* = 6/group) mice were immunized at week 0 with either empty DNA vector (DNA-psv) or S-expressing DNA vector (DNA-psv-S), followed by booster immunization at week 4 with DNA-psv-S, parental K562 cells (K562-WT), or K562-S cells that were untreated (K562-S) or through inactivation by formalin (K562-S-FI) or X-ray irradiation (K562-S-X-ray). The dosages of DNA vectors and K562-S were 100 μg and 1 × 10^6^ cells, respectively. (B and C) Serum antibody responses were assessed at week 6 post prime by a RBD-specific binding antibody ELISA (B) and pseudovirus neutralization assays (C). (D and E) Assessment of Th1 or Th2 bias in the immune response. The serum levels of IgG2c and IgG1 antibody were determined at week 6 post prime by ELISA (D) to calculate the IgG2c/IgG1 ratio (E), which is a reliable surrogate measure of Th1/Th2 status. Antibody titer data were presented as geometric mean titers (GMT) ± geometric standard deviation (GSD). Kruskal–Wallis test with Dunn’s adjustment were applied when comparing groups. ns = no significant.

Given K562 cells being a human erythroleukemic cell line, inactivation of K562-S cells is an essential step before their administration as a vaccine. Thus, we examined in a mouse model, the impact of different inactivation protocols on the immunogenicity of K562-S. To this end, we inactivated K562-S cells by either X-ray irradiation or treatment with formalin (FI) and administered them to C57/BL6 mice in comparison to untreated K562-S cells in a 4-week two-shot regimen consisting of S-encoding DNA vaccine (DNA-psv-S) as prime and K562-S as boost (Figure 2(A)). Our adoption of this regimen for evaluating the immunogenicity of live or inactivated K562-S was based on many vaccination regimens in the literature, in which DNA is used to prime responses that are then boosted by another type of vaccine [22,23]. The three groups of animals were named, respectively, as K562-S, K562-S-Xray, K562-S-FI according to the identity of the boost. Sera were subsequently collected at week 6 post prime for determination of anti-RBD antibody titers by ELISA and neutralizing titers by pseudovirus inhibition assay. The three vaccination groups showed comparable anti-RBD IgG endpoint titers (Figure 2(B)) and pseudovirus ID50 titers (Figure 2(C)), suggesting minimal effect of both inactivation methods on the immunogenicity of K562-S. In contrast, neither of titers could be detected in K562-WT group. We further examined whether inactivation affects other aspects of K562-S-induced immune response. Previous studies on SARS vaccines indicated that induction of a Th1-biased response is critical for the success of a vaccine as a Th2-biased immune response was often linked to increased risk of vaccine-enhanced respiratory disease (VAERD). The ratio of IgG1 to IgG2a/c is widely used as a surrogate of Th1:Th2 polarization, and we consequently analyzed the profiles of RBD-specific IgG-subclass in sera collected at week 6 post prime. The analyses revealed that IgG2c to IgG1 ratio was close to 1 for both the live K562-S group and K562-S-Xray group while slightly less than 2 for K562-S-FI group, consistent with a balanced T cell response (Figure 2(D,E)).

For further evaluation of T cell responses, spleen cells were isolated from immunized mice at week 6 (*n* = 6 per group), and their production of IFN-γ was measured by ELISpot assay after stimulation with pools of RBD peptides. The peptide pools were designed by dividing the RBD sequence into 13 pools, each containing five peptides of 15 amino acids length with 11 amino acids overlap (Figure S2A). Similar to the antibody responses, the total T cell responses to the RBD peptide pools, as reported in spot-forming units per 10^6^ splenocytes, were comparable among the three K562-S immunization groups (Figure S2B). In addition, the analyses of T cell responses to individual peptide pools revealed that, for all three groups, the strongest response was detected to peptide pool 11 (Figure S2C). We further assessed the response to individual peptide included in the peptide pool 11 and observed a focused response on peptide 54 and 55, suggesting the shared 11 amino acids likely represents the most dominant epitope for K562-S-elicited T cell immunity (Figure S2D). Given that chemical-mediated inactivation is currently the general method used in industry for the production of inactivated virus vaccine, we chose FI-inactivated K562-S, briefed as K562-S-FI, as the vaccine for further investigation.

### Enhancing effect on immune response to K562-S vaccine by adjuvants

Next, we determined whether the K562-S vaccine-induced antibody response can be further enhanced using adjuvants [24]. To this end, we first explored the effect of dosage on the immunogenicity of K562-S-FI administered with traditional Alum adjuvant. We tested four different dosages (2E4, 1E5, 5E5, and 1E6 cells per dose, respectively) within a two-shot regimen, and sera were collected at week 6 after the second shot for RBD-based ELISA and pseudovirus neutralization assay (Figure S3A). In terms of RBD-specific antibodies, similar levels were detected among the four dosage groups but the three higher dosage groups consistently showed lower within-group difference as compared to the 2E4 dosage group. In contrast, the average titers of neutralizing antibody appeared to be positively correlated with the dosage, with a 11-fold increase from 2E4 group to 1E6 group observed at week 6 (Figure S3B,C). Using sandwich ELISA, we estimated that 1E6 K562-S cells had an S antigen dose of approximately 0.47 ug (Figure S3D). As 1E6 dosage affords the highest neutralizing activity, we chose this dosage for further vaccine optimization.

**Figure 3.**
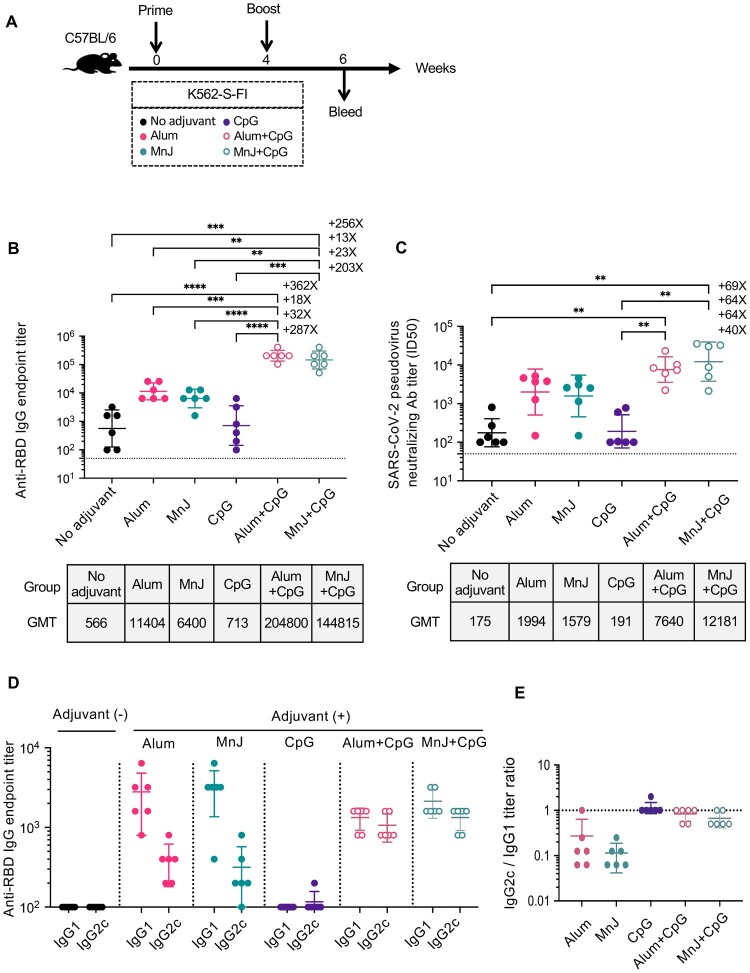
Identification of optimal adjuvant for K562-S vaccine in induction of humoral immunity against SARS-CoV-2. (A) Experimental schedule. Female C57/BL/6 (*n* = 6/group) mice were subjected to a homologous prime-boost regimen with 4-week interval consisting of K562-S-FI either non-adjuvanted or adjuvanted with Alum, MnJ, CpG, Alum plus CpG, or MnJ plus CpG. (B and C) Serum antibody responses were assessed at week 6 post prime by RBD-specific binding antibody ELISA (B) and pseudovirus neutralization assays (C). (D and E) Serum levels of SARS-CoV-2 RBD-specific IgG1 and IgG2c antibodies as determined by an ELISA (D) and the derived IgG2c/IgG1 ratios (E). Antibody titer data were presented as geometric mean titers (GMT) ± geometric standard deviation (GSD). Kruskal–Wallis test with Dunn’s adjustment were applied when comparing groups. * *p* < 0.05, ** *p* < 0.01, *** *p* < 0.001; **** *p* < 0.0001, ns = non-significant.

We used the same two-shot regimen as shown above for immunologic evaluation of several other adjuvants, alone or in combination, for us with K562-S-FI (Figure 3(A)). Sera collected at week 6 were used for determination of S-specific antibody and cellular responses. The combinatorial use of K562-S-FI with either Alum or MnJ resulted in 11–20 fold increase in geometric mean titers (GMT) of RBD-specific binding antibody as well as 9–11 fold increase in neutralizing antibody, as compared to the non-adjuvant control. In contrast, there was no enhancing effect detected for CpG adjuvant. However, the addition of CpG to the formulation could further potentiate the enhancement of antibody response by Alum or MnJ, resulting in an additional 18–23-fold increase in GMTs of RBD-specific binding antibody and 4–8-fold increase in neutralizing antibody. As such, all the animals in Alum or MnJ plus CpG group displayed a high neutralizing antibody titer of >2000 (Figure 3(B,C), Supplementary Figure 7A). We further evaluated the adjuvant effect on the Th1/Th2 balance, assessed by the ratio of IgG1 to IgG2c [25,26]. The results showed that Alum or MnJ adjuvant drove the K562-S-FI-induced immunity toward a Th2-biased phenotype whereas their combination with CpG reversed Th2 biasing and induced a balanced T cell response (Figure 3(D,E)). Surprisingly, quantification of T cell responses to RBD peptide pools by IFN-γ ELISpot assay revealed a negative relationship between T cell response and antibody responses. Non-adjuvant group displayed the highest frequency of IFN-γ-secreting cells whereas little difference was detected between the four adjuvant groups and control group, indicating ineffective activation of T cell responses (Figure S4A). Taken together, among the adjuvants we tested, Alum or MnJ plus CpG delivered the best performance, evidenced by higher magnitude neutralizing antibody response and a more favourable Th1/Th2 balance.

### Impact of storage condition on the efficacy of K562-S-FI vaccine in mice

We assessed the effect of storage conditions on the stability of K562-S vaccines. Using the DNA-S prime/K562-S boost regimen to vaccinate mice, we found cryopreservation (>4 weeks) has a marginal effect on the efficacy of K562-S-FI vaccine in terms of both anti-RBD IgG and neutralizing antibody titers (Figure S5A-C). K562-S-FI vaccines also showed sustained efficacy in inducing antibody response after being stored at RT for 1–2 months when tested as both prime and boost in a two-step regimen (Figure S5D-F). These results support K562-S-FI having a favourable stability property to meet the needs of a successful vaccine.

### K562-S vaccination-mediated protection against SARS-CoV-2 challenge in hACE2 transgenic mice

**Figure 4.**
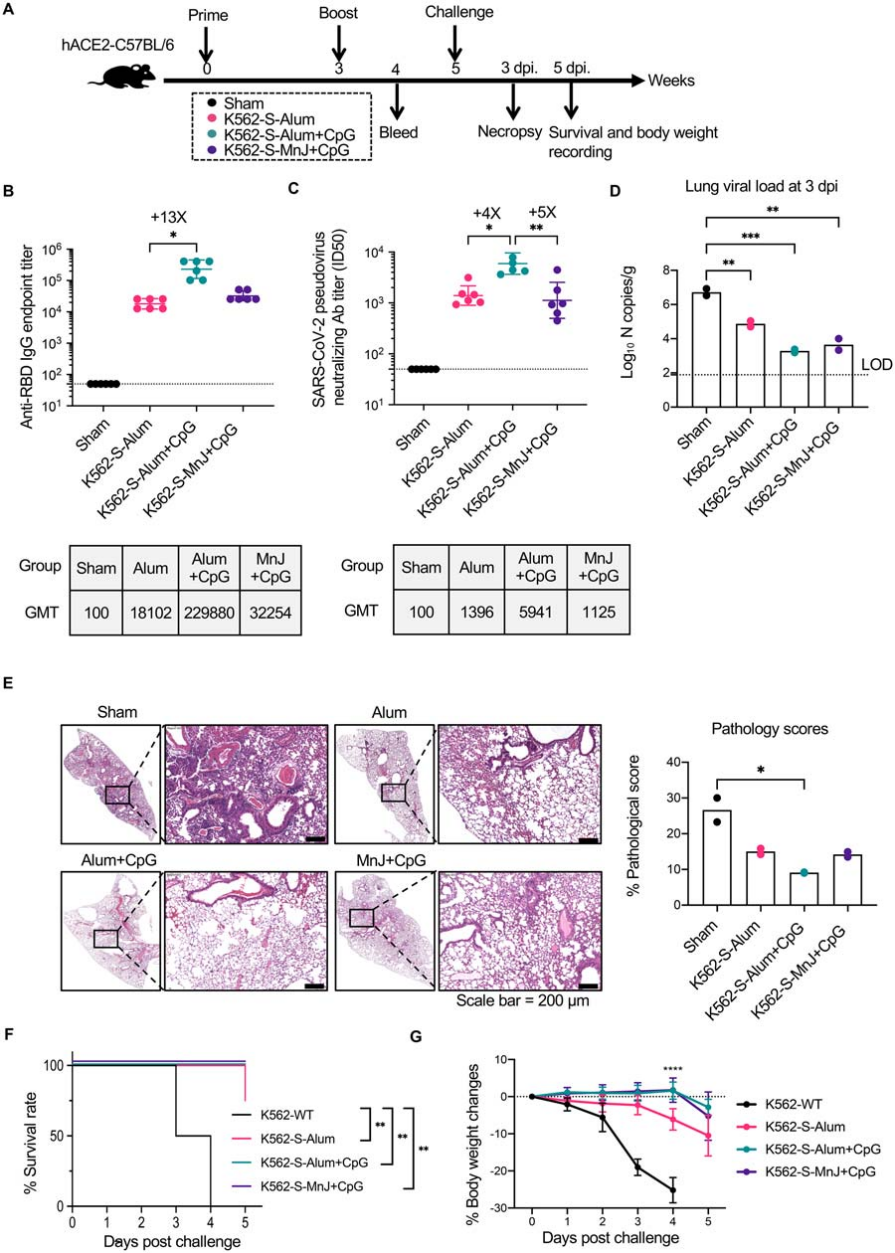
Immunogenicity of K562-S vaccines and afforded protection against the SARS-CoV-2 in hACE2 mice. (A) Experimental schedule. Transgenic hACE2-C57/BL/6 mice (*n* = 6/group) were vaccinated intramuscularly (IM) with 1 × 10^6^ cells of alum adjuvanted parental K562 (sham) or indicated adjuvanted K562-S at week 0 (prime) and 3 (boost), followed by intranasal administration of 1 × 10^4^ PFU of SARS-CoV-2 (CHN/Shanghai_CH-02/2020) at week 5. (B and C) Serum antibody responses were assessed at week 4 post prime by RBD-specific binding antibody ELISA (B) and pseudovirus neutralization assays (C). (D) Viral RNA copies in the lungs on day 3 post infection. RNA was extracted from lung homogenates; the contained viral N transcripts were determined by qRT-PCR and plotted as log10 copies per gram (tissue weight). (E) Representative images of H&E-stained lung sections from sham and vaccination mice on day 3 after SARS-CoV-2 infection. Lung histology data were used to calculate quantified pathological scores. Scale bar, 200 μm. Results are expressed as mean ± SD. (F) Kaplan-Meier survival curves of challenged mice were compared by using log-rank test and (G) the body weight changes were plotted for each group. Data are means ± SD.. Comparison among groups were conducted by Kruskal–Wallis test with Dunn’s adjustment. *p < 0.05; **p < 0.01; ***p < 0.001; ****p < 0.0001; ns, no significant.

We used hACE2 transgenic mice to examine the protective efficacy of K562-S vaccine against SARS-CoV-2. To this end, hACE2 transgenic mice were intramuscularly vaccinated by a two-shot, 3-week interval regimen using K562-S vaccine in combination with a different adjuvant as both prime and boost, or sham-vaccinated with Alum-adjuvated wild-type K562 (*n* = 6 per group) (Figure 4(A)). The animals were first analyzed at 1 week post boost for the serum antibody response. Although all four K562-S-vaccinated groups showed significant induction of RBD-specific antibody response compared to the sham control, Alum plus CpG formulation yielded the highest level in both anti-RBD binding and neutralizing antibodies with GMT of 229,880 and 5961 titers, respectively. In comparison, the Alum group showed an approximately 13-fold less in GMT of anti-RBD binding antibody and 4-fold decrease in GMT of neutralizing antibody, similar to the MnJ plus CpG group (Figure 4(B,C)). Thus, two doses of K562-S vaccines were effective in eliciting potent neutralizing antibody response, particularly when adjuvated with Alum and CpG.

Subsequently, all mice were challenged with 10,000 PFU viruses of SARS-CoV-2 via intranasal route to evaluate the protective efficacy of K562-S vaccines. As our previous experience with hACE2 transgenic mice as a SARS-CoV-2 infection model informed that the viral burden in the lung reached peak at 3 days post infection (dpi), lungs were harvested at 3 dpi for the assessment of viral loads and histopathology. The lung viral loads, measured as the copies of viral N genes per gram by quantitative RT–PCR assay, were correlated with the magnitude of antibody responses. While the average viral load of the sham group was determined to be 10^6.7^ copies/g, the Alum plus CpG group, which was endowed with the highest antibody response, displayed nearly 3-log reduction in viral load relative to sham group (Ct value: Alum plus CpG = 29, sham = 17), in contrast to the approximately 2-log reduction observed in the Alum group (Ct value: Alum = 25, sham = 17). Interestingly, the MnJ plus CpG group achieved a similarly lower viral load as seen with the Alum plus CpG group (Ct value: Alum plus CpG = 29, MnJ plus CpG = 27) (Figure 4(D)). K562-S-vaccinated mice also demonstrated significant amelioration in lung pathology as compared to sham control, with a significantly lower pathology score justified by minimal bronchiolar epithelium disruption and markedly reduction in inflammatory infiltrates (Figure 4(E)). We also monitored the survival and body weight changes after SARS-CoV-2 challenge. In contrast to the sham group, which showed 100% mortality by 4 days post infection (dpi), animals from K562-S-Alum + CpG and K562-S-MnJ + CpG groups all survived during the 5-day recording period with only moderate (∼5%) reduction in body weight. K562-S-Alum group exhibited less protection with one death and an average decrease of approximately 10% in body weight by 5 dpi (Figure 4(F,G)). Collectively, these data demonstrated that K562-S vaccines can afford effective protection against SARS-CoV-2 infection.

**Figure 5.**
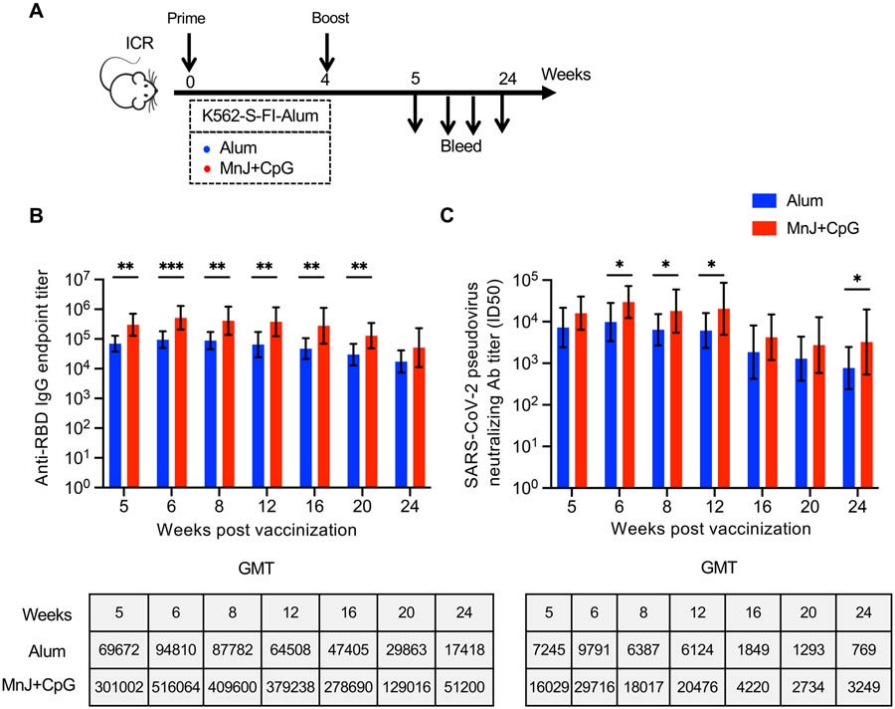
Strength and durability of antibody response to K562-S vaccination in a different mouse strain. (A) Schedule of vaccination and specimen collection. Female ICR (*n* = 9/group) mice were subjected to a homologous prime-boost regimen with 4-week interval consisting of either alum adjuvanted K562-S-FI or MnJ plus CpG adjuvanted K562-S-FI. Sera were collected between week 5 and 24 post prime for antibody assessment. (B and C) Time-course changes in RBD-specific binding antibody titers as determined by ELISA (B) and neutralizing antibody titers as assessed against pseudovirus (C). The tops of the bars represent the geometric mean titers (GMTs). Significance of difference between means was assessed by nonparametric Mann–Whitney test. *p < 0.05; **p < 0.01; ***p < 0.001; ****p < 0.0001; ns, no significant.

It was previous reported that the same vaccine might display different efficacy in different mouse strain. Thus, we examined the efficacy of K562-S vaccine in another mouse strain, namely ICR strain mice, using either Alum or MnJ plus CpG adjuvanted K562-S-FI vaccines as both prime and boost in a four-week prime-boost regimen (Figure 5(A)). The sera were collected at varied time points up to 24 weeks from the start of vaccination for analyzing antibody responses. Consistent with the results seen in C57/BL6 strains, MnJ plus CpG group had higher GMTs of anti-RBD antibody and neutralizing antibody than Alum group at each time point. The comparison of the GMT titers at 2 week post boost between C57/BL6 and ICR mice indicated the later responded more strongly, with 8.3- and 3.6-fold increase in anti-RBD antibody titers for Alum and MnJ plus CpG group, respectively, in line with the corresponding 4.9- and 2.4-fold enhancement of neutralizing antibodies titers (Figure 5(B,C), Supplementary Figure 7B,C). For both vaccination groups, the anti-RBD antibody response started to decrease between week 16 and 20 and declined slowly afterwards, while the neutralizing antibodies displayed a relatively earlier and sharper decline initiating between week 12 and 16 before appearing to reach a plateau. However, the neutralizing titers at week 24, despite decreasing 4.9–9.4-fold relative to week 5, remained at high level, with GMT being 769 and 3249, respectively for Alum and MnJ plus CpG groups (Figure 5(B,C) Supplementary Figure 7B,C). Thus, K562-S-FI generally showed a strong antigenicity in mouse models, and was able to elicit a potent neutralizing antibody response lasting for at least 5 months.

### Evaluation of K562-S-FI vaccines in non-human primate animal model

**Figure 6.**
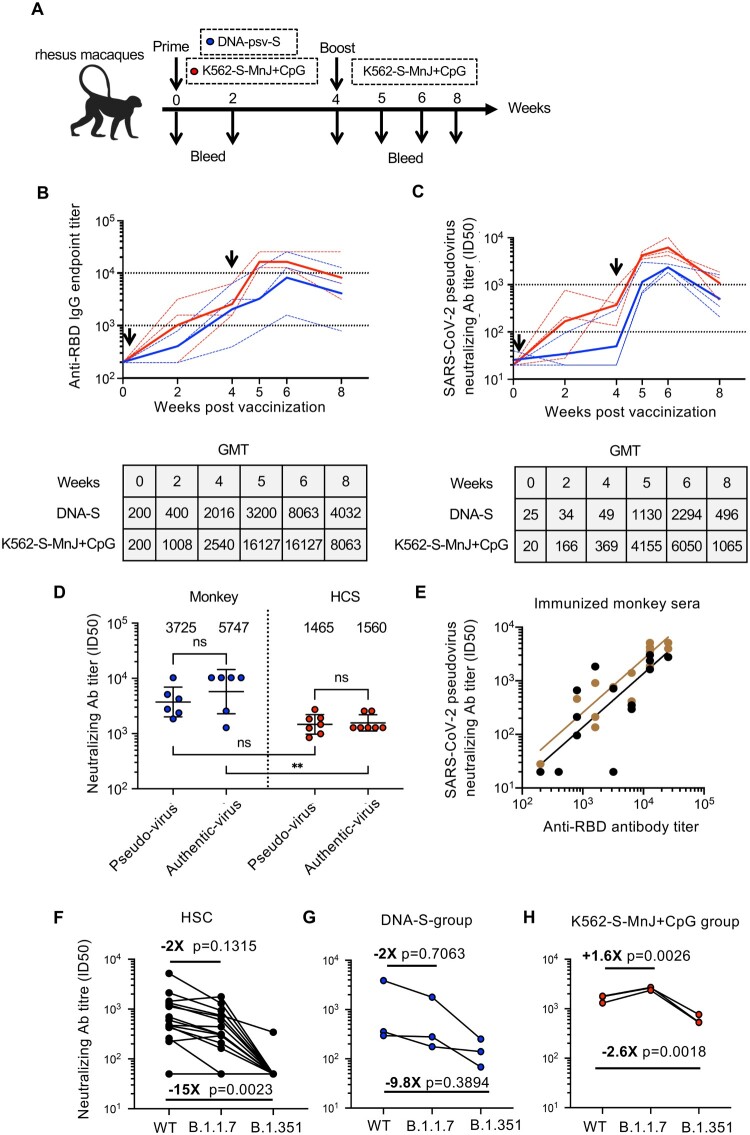
Immunogenicity of K562-S vaccines in non-human primates. (A) Schedule of vaccination and specimen collection. Rhesus macaques were primed with 1 mg of DNA-S or 1 × 10^7^ MnJ plus CpG adjuvanted K562-S-FI cells at week 0, and boosted with 1 × 10^7^ MnJ plus CpG adjuvanted K562-S-FI cells at week 4. Sera were collected at week 0, 2, 4, 5, 6, and 8. (B and C) Time-course changes in RBD-specific binding antibody titers as determined by ELISA (B) and neutralizing antibody titers as assessed against pseudovirus (C). The dotted lines represented data collected from individual animals, coloured differently according to the prime: DNA-S, blue; adjuvanted K562-S, red. The solid line showed the trend of GMTs. (D) Comparison of neutralizing antibody titers, as determined by both pseudovirus and authentic SARS-CoV-2 neutralization assays, between vaccinated monkey sera and human convalescent sera (HCS). The assayed monkey sera were collected at week 6 post prime from all six vaccinated animals. The GMT values were shown on top of the plot. Significance of difference between means was assessed by nonparametric Mann–Whitney test. **p < 0.01; (E) Correlation analysis between RBD-specific binding antibody titers and pseudovirus neutralizing antibody titers. Trends lines were separately created for DNA-S prime group (black) and K562-S prime group (brown) and the data were used for calculation of Spearman correlation coefficients, which were indicated adjacent to the lines. (F–H) Neutralization titers against SARS-CoV-2 versus its variants B.1.1.7 and B.1.351 in human convalescent sera (HCS) (F), sera from DNA-S group (G), and sera from K562-S-MnJ + CpG group (H). The pairwise mean-fold changes in titers were indicated. Significance of difference was assessed by Kruskal–Wallis test with Dunn’s adjustment.

Built on the above experience with vaccination in mice, we tested the efficacy of K562-S-FI vaccine in non-human primates. To this end, six rhesus macaques were divided into two groups and subjected to 4-week vaccination regimen administered via intramuscular route with either DNA-S or MnJ plus CpG adjuvanted K562-S-FI (K562-S-MnJ + CpG) as prime and K562-S-MnJ + CpG as boost. According to the identity of priming, the two groups were, respectively, abbreviated as DNA-S group and K562-S-MnJ + CpG group. The sera were collected at the time of vaccination (week 0) and various time points after vaccination up to week 8 for the determination of serum antibody responses (Figure 6(A)). In terms of anti-RBD binding antibody response, although the GMT for DNA-S group was 2.5-fold lower than K562-S-MnJ + CpG group at week 2, GMTs continued to increase and attained a similar level for both groups at week 4 (Figure 6(B)). K562-S-MnJ + CpG priming was also effective in eliciting neutralizing antibodies, attaining a GMT of 166 and 369 at week 2 and 4, respectively, whereas the corresponding GMT values for DNA-S were only 34 and 49, just slightly above the starting GMT of 20–25. The poor activation of neutralizing antibody response of DNA-S was significantly resolved by the followed K562-S-MnJ + CpG boosting, resulting in approximately 23-fold increase in GMT to reach 1130 one week after boost, though still 3–4-fold lower than corresponding GMT for K562-S-MnJ + CpG group. Both vaccination groups achieved peak values of neutralizing antibody titer at week 6 (GMT of 6050 for MnJ plus CpG group vs. 2294 for DNA-S group) and then showed a decline afterwards; the GMTs for DNA-S and K562-S-MnJ + CpG group at week 8 remained high at 496 and 1065, respectively (Figure 6(C), Supplementary Figure 7D).

Considering the importance of human translation of the immune response in our NHP model, we compared the vaccinated monkey sera to HCS. To this end, sera collected from the six vaccinated monkeys at week 6 and seven serum samples from recovered CoV-19 patients were analyzed in parallel for antibody responses. The measurements from pseudovirus neutralization assay showed that the GMT for neutralizing antibodies for monkey sera was 3725, approximately 2.5-fold higher than HCS. This difference was confirmed in second neutralization assay with authentic SARS-CoV-2 virus, which yielded slightly higher GMTs for both monkey vaccinated sera and HCS samples (Figure 6(D), Supplementary Tables 2 and 3). We also measured the anti-RBD antibody titer and consequently evaluate its relationship with pseudovirus neutralizing antibody titer. A positive linear correlation was observed between the two titers, with pseudovirus neutralizing antibody titers being matched to approximately one-tenth of RBD-specific binding antibody titers (Figure 6(E)). Thus, K562-S vaccine demonstrated efficacy in the induction of robust neutralizing antibody response in NHP, further supporting its future clinical exploration as a novel SARS-CoV-2 vaccine candidate.

The newly emerged SARS-CoV-2 variants, B.1.1.7 in the United Kingdom and B.1.351 in South Africa, have aroused increasing concern as they may escape immunity established by previous infection or vaccination [27]. We thus also assessed the reactivities of sera from the above K562-S-vaccined monkeys to these variants relative to wild-type SARS-CoV-2 by analyzing neutralization against corresponding pseudoviruses, with convalescent serums (HCS) included as control. In HCSs, the neutralizing antibody GMT against B.1.1.7 and B.1.351 variants decreased, respectively, by approximately 2-fold and 15-fold relative to wild-type (WT) (Figure 6(F)). As for vaccinated sera from DNA-S group, the relative neutralizing activities to a B.1.1.7 and B.1.351 as compared to WT was dropped by 2 and 9.8 fold, respectively, whereas in sera from K562-S-MnJ + CpG group, the B.1.351 neutralization showed a moderate 2.6 fold decrease while the B.1.1.7 neutralization was even more robust. These results were consistent with previous studies on HCSs or serum specimens obtained from recipients of mRNA, inactivated and protein subunit vaccines, showing the stronger immune escape ability of B.1.351 than B.1.1.7 (Figure 6(G,H)) [27–29]. Interestingly, we observed that, between the two immunization groups, K562-S-MnJ + CpG group showed a neutralizing activity less affected by B.1.351 variant, further demonstrating the superiority of the two-shot regimen employing only MnJ + CpG adjuvanted K562-S vaccine.

A major safety concern of K562-S vaccine is the potential induction of autoantibody against its cellular components, especially nuclear proteins. To evaluate this concern, we measured the immune responses against K562 cellular proteins, including whole-cell lysate of K562-WT (K562-WT-lysis) and CD71, an erythroid precursors marker highly expressed in K562-WT), in K562-S immunized monkey, and compared them to those in sera of immunized mice. As shown in Figure S6, in mouse serum, the binding titers of K562-WT-lysis were significantly lower than those specific for CD71, consistent with the identity of K562-S as a whole-cell vaccine. Importantly, for both CD71 and K562-WT-lysis, monkey sera showed much lower reactivities than mice sera, indicating diminished vector effect in animals closely related to human. On the basis of these results, we inferred a negligible immune response against K652-inherent cellular proteins being raised by K562-S vaccination in human settings.

## Discussion

In this study, we introduced a human cell-based new platform for the development of SARS-CoV-2 vaccine. We constructed the first of its kind, namely K562-S, through stable transfection of S-protein-expressing gene into K562 cells followed by clonal selection. A unique advantage of K562-S over previously developed SARS-CoV-2 vaccines is the presentation of S antigen in its native-like, membrane form, which would be expected to increases the likelihood of eliciting a protective immunity resembling that raised by natural infection. Our subsequent investigations further revealed several properties of K562-S that are important prerequisites for a human vaccine, including: (1) K562-S is highly immunogenic, capable of eliciting both humoral and cellular responses against S-protein; (2) inactivation of K562-S by either X-ray irradiation or formalin (FI) does not compromise its immunogenicity; (3) the efficacy of K562-S is well preserved after freezing or long-term room temperature storage. We also conducted studies on how the immunogenicity of K562-S was impacted by co-administration with specific adjuvants, including Alum, MnJ and CpG, individually and in combination. MnJ is a novel adjuvant derived from the discovery that Mn2+ is a potent inducer of type I interferons by activating cGAS-STING pathway, and was evidenced recently for its capability to stimulate CD4+/CD8+ T cell proliferation, indicating a potential to facilitate antibody production [30–32]. Consequently, we identified a dual-component adjuvant, CpG plus Alum or MnJ, as an effective adjuvant to augment the antibody response elicited by K562-S while driving the response toward a Th1/Th2 balance. Built on these findings, we developed a two-dosage, four-week interval regimen using adjuvated, formalin-inactivated K562-S and examined its protective efficacy in hACE2 transgenic mice against SARS-CoV-2 challenge. The vaccinated mice demonstrated induction of high levels of anti-RBD binding antibodies and neutralizing antibodies, underlying significant protection from SARS-CoV-2 challenge, characterized by 1000-fold reduction in viral loads and improvement in lung pathology as compared to the sham control. Importantly, despite undergoing attrition, K562-S-afforded serum neutralizing activity was well maintained for at least 5 months in mice. We further showed the same regimen was effective in rhesus macaques as it was capable of inducing authentic neutralizing antibody to a level 3-fold higher than human convalescent sera, suggesting the possibility of successful translation to human clinical trials.

K562-derived whole-cell vaccine approach has been previously explored for cancer treatment by engineering K562 to express immune-regulatory cytokines, and more recently tumour antigen [17–19]. Such exploration established the safety of inactivated K562-derived vaccine for human use. Our results, showing that inactivation did not compromise the immunogenicity of K562-S and inactivated K562-S vaccine retains activity following long-term cryopreservation and room temperature storage, strengthened our view that K562-S can be developed as a novel vaccine against SARS-CoV-2. One surprising finding emerged from our characterization of combining K562-S-FI with different adjuvant is that adjuvant can dramatically alter the immunity raised by K562-S-FI. In terms of neutralizing antibody, combination with Alum or MnJ can significantly potentiate the efficacy of K562-S-FI with a Th2 bias. The addition of CpG to the formulation was able to further augment the antibody response while switching the immunity from a Th2-skewed response to a balanced Th1/Th2 response representing more favourable safety profile. However, there was a tradeoff of such adjuvant-mediated enhancement of antibody responses, that is, a marked drop in cellular responses. Currently, we did not know the exact mechanism by which K562-S engages the immune cells. It has been postulated that K562 cells are a favourite target of natural killer (NK) cells because of its lack of expression of MHC cells [14]; the NK-induced K562 cell death promotes cytokine release and consequently the recruitment of antigen presenting cells (APCs) to the vaccination site and their maturation, which couples with the concomitant releasing of the contents of K562, including the expressed antigen, to engage immune effector cells for mounting antigen-specific immune responses [16]. It will be of interest to evaluate other adjuvants to see if they can assist K562-vaccine to achieve both a strong Th1-biased antibody response and an effective cellular immunity.

To date, a variety of approaches have been attempted to develop a SARS-CoV-2 vaccine, ranging from traditional approaches represented by whole inactivated virus and protein subunit vaccines to novel strategies epitomized by viral vector, DNA, and mRNA-based vaccines [25,33–39]. Each approach has its unique advantage(s) and limitation(s). For instance, the whole inactivated vaccine approach appeared to be less effective in inducing neutralizing antibodies as compared to some of other approaches, whereas the replication-defective adenoviral vector vaccines, especially those based on adenovirus type 5, might face the challenge of pre-existing immunity to the vaccine viral carrier. Protein subunit vaccine with adjuvants prevail in immune response induction, but for manufacturing, proper purification technology needs to be developed to maintain conformation in natural form. The advantages of mRNA vaccines lie in their superior immunogenicity and preparedness-theoretically only the immunogen sequence is needed, however, their global application has been affected by supply logistics issues particularly for BNT162b2 (Pfizer-BioNTech), which requires ultra-cold storage to maintain the activity, let alone the need to be encapsulated in lipid nanoparticles for formulation [33].

Despite the acknowledgement that it is nearly impossible to compare the efficacy of different vaccines because of different methods and reagents being used for respective evaluation [40], the comparison between the immunogenicity data of K562-S and those of vaccines currently in deployment for vaccinating the human population might give a sense of the value of K562-S. Time-course analysis revealed that the peak pseudovirus neutralizing GMT induced by two doses of MnJ plus CpG adjuvated K562-S-FI in ICR mice was reached two weeks after the boost at 29716, in comparison to 12181 attained in C57BL/6 mice at the same time point with the same regimen. Among the available SARS-CoV-2 vaccine platforms, the mRNA platform appeared to afford the greatest immunogenicity. Two doses of 1 μg of mRNA-1273 vaccine could induce pseudovirus neutralizing GMTs up to 819 in BALB/c mice and only 89 in C57BL/6 mice [39]. Thus, if there exists comparability between pseudovirus neutralizing assays, the data comparison would indicate a better performance of 1 × 10^6^ of K562-S cells than 1 μg of mRNA-1273 in inducing neutralizing antibodies. A similar difference was also observed in vaccinated rhesus macaques as two doses of 100 µg mRNA-1273 vaccine attained robust neutralizing antibody response with live-virus GMTs of 3481 4 weeks after the second vaccination, which is lower than the live-virus GMTs of 6050 achieved by two doses of 1 × 10^7^ of K562-S cells [41]. However, when human convalescent sera (HCS) were used as the reference, the order seems reversed as the neutralizing titers induced by 10 µg and 100 µg mRNA-1273 were, respectively, 12 and 84 times as high as those in HCSs whereas in our measurements K562-S-induced serum neutralization titers was on average 3.7 higher than values exhibited by HCSs. Comparisons of vaccination-induced serum neutralization titers with HCSs were also reported for other types of SARS-CoV-2 vaccines. For example, the serum neutralization titers induced by an S-protein subunit vaccine adjuvated with Matrix-M (NVX-CoV2373) in macaque was 7.9–10.1 fold higher than those by HCSs [42], whereas the sera from traditional inactivated virus vaccine (PiCoVacc) immunized macaque exhibited similar neutralizing activities as compared to HCSs [39]. The deduction of the relative potency of different vaccines by using HCS was confounded by the fact that different HCS might have different levels of neutralizing antibodies; sera samples we used in this study were mainly derived from prolonged hospitalized patients, who might develop higher neutralization antibody responses. Taken together, we conclude that the ability of engaging antibody response shown by K562-S vaccine in animal vaccinations supported its qualification of a human COVID-19 vaccine candidate.

Antibody-dependent enhancement (ADE) and vaccine-associated enhanced respiratory disease (VAERD) are considered as potential concerns of SARS-CoV-2 vaccine. ADE was first identified for dengue infections and later extended to multiple viruses including SARS-CoV and MERS-CoV [43,44]. The current theory of ADE attributes non-neutralizing antibodies or antibodies at sub-neutralizing levels to the cause of ADE as the binding of antibody to the newly infecting virus does not result in neutralization but instead promote the virus uptake of host cells via Fcγ receptors (FcγRs) [3]. On the other hand, VARED, which was observed in mouse model with some of the inactivated vaccines and particularly experimental SARS-CoV vaccines, has been associated with Th2-biased immune response [42–45]. We have not found any K562-S-vaccinated hACE2 transgenic mice, upon SARS-CoV-2 challenge, showing more aggravated phenotypes including viral load, weight change, and mortality compared to sham control mice. This observation would suggest that K562-S vaccination was highly unlikely to induce ADE or VARED effect, which was in line with its ability to engage a balanced Th1/Th2 immune response featuring high neutralizing antibody titers. K562-S vaccination also demonstrated excellent durability of efficacy in ICR mice, with high titers of anti-RBD binding antibodies and neutralizing antibody lasting at least 5 months. Thus, favouring a persistent and balanced antibody response added further credence to the promise of K562-S as an effective CoV-19 vaccine.

Lastly, as the first whole human cell-based vaccine against CoV-19, K562-S can be modified to improve its potency and breadth. A clear advantage of human cell-based vaccine over traditional vectored vaccine such as protein subunit and inactivated virus vaccines is that the former has unmatchable carrier capacity, meaning less constraints on the size of grafted exogenous sequences. Thus, K562-S can be easily further engineered by the introduction of S genes from SARS-CoV-2 variants [46], SARS-CoV and/or MERS-CoV to make it a universal coronavirus vaccine, or immune-modulatory molecules to increase the engagement of immune cells or combining both. K562-S also has several concerns. The first concern is its safety, which stems from the identity of K562 as a human leukemic cell line. We addressed this concern by demonstrating the high effectiveness of formalin fixation (also UV irradiation) in killing K562 cells, consistent with previous studies. Further evidence was provided by ongoing clinical trials of K562/GM-CSF, where only mild local reactogenicity was observed following subcutaneous (SC)/intradermal (ID) injection of K562/GM-CSF cells. Nevertheless, we acknowledge that, in future exploration of K562-S vaccine in human trials, additional measures such as exposing the cells to lethal freezing temperature after formalin fixation should be taken to ensure complete cell killing. Another major concern comes from vector effects of K562-S that might result in the induction of antibody against K562 cellular and surface proteins. However, despite the concerned effect being observed in K562-S-vaccinated mice, it was greatly diminished in vaccinated macaque, indicating that the chance for K562-S causing vector effect in the human setting would be extremely low. Finally, like other currently available vaccines, K562-S showed decreased efficacy against the emerging B.1.351 variant compared to wild type but this decrease was approximately only two-fold. We envision constructing multivalent vaccine(s) simultaneously displaying wild type and its variant(s) such as B.1.351 and exploring its human use following the two-shot regimen with Alum/MnJ plus CpG as adjuvant would be the next step to harness the potential of whole human cell-based vaccine to fight SARS-CoV-2 pandemics. It is also should be noted that adjuvanted K562-S, demonstrating its ability to boost immune responses primed by DNA vector, might find utility in a heterologous prime-boost regimen with different types of vaccine that have already been deployed in human populations.

## Limitations of study

The major limitation of our study stems from the major challenge of evaluating a human cell-based vaccine before clinical trials. The immunogenicity and protective efficacy of K562-S in the mouse model may be influenced by the graft-versus-host effect associated with using a human cell carrier. However, we would expect the observed K562-S-induced antibody responses to be dispersed on all the membrane proteins on K562-S cells that mice see non-itself, thus hampering the focusing of immune system on the S-protein. Indeed, we observed similar if not higher magnitude neutralizing antibody responses in the rhesus macaque, which is among the most closely related animal models of human. Due to time and resource constraints, we have not tested the protective efficacy of our two-shot K562-S vaccination program in rhesus macaque, which may provide the strongest testimony for its translation to human setting. However, based on the robust neutralizing titer displayed by the vaccinated animal, which was even higher than those reported for mRNA vaccine, it is reasonable to speculate that our K562-S regimen would afford effective protection against SARS-CoV-2 challenge in rhesus macaque model. Another limitation of our study is that we only track the antibody responses to K562-vaccine in rhesus macaque for a short 4-week period after the boost, where the neutralizing titer started to show a decline between week 6 and 8 (the starting week of vaccination as week 0). A similar decline was also observed with mRNA-1273 vaccine when administered in monkeys [41]. We plan to conduct a longitudinal investigation of the durability of the K562-S-induced antibody response in the future, which will help determine whether a second boost of K562-S or other types of vaccine is required for human use.

In summary, we developed a new SARS-CoV-2 vaccine candidate in human K562-derived whole-cell vaccine expressing viral S-protein in membrane form and presented strong evidences from both mouse and non-human primate models supporting its efficacy in inducing neutralizing antibodies and virus protection as well as its feasibility. We hope that K562-S vaccine and its improved version of future will be a valuable addition to our vaccine arsenal against threats from pathogenic coronavirus, including but not limited to SARS-CoV-2. Critically, this platform provides a novel approach for rapidly developing new vaccines, including multiple-valent ones.

## Supplementary Material

Supplemental Material

## Data and materials availability

All data are available in the main text or the supplementary materials.
