# Supplementary figures and images for: A human cell-based SARS-CoV-2 vaccine elicits potent neutralizing antibody responses and protects mice from SARS-CoV-2 challenge
Source: Emerg Microbes Infect. 2021 Aug 12;10(1):1555–73. doi: 10.1080/22221751.2021.1957400 (PMC8366622; doi:10.1080/22221751.2021.1957400)

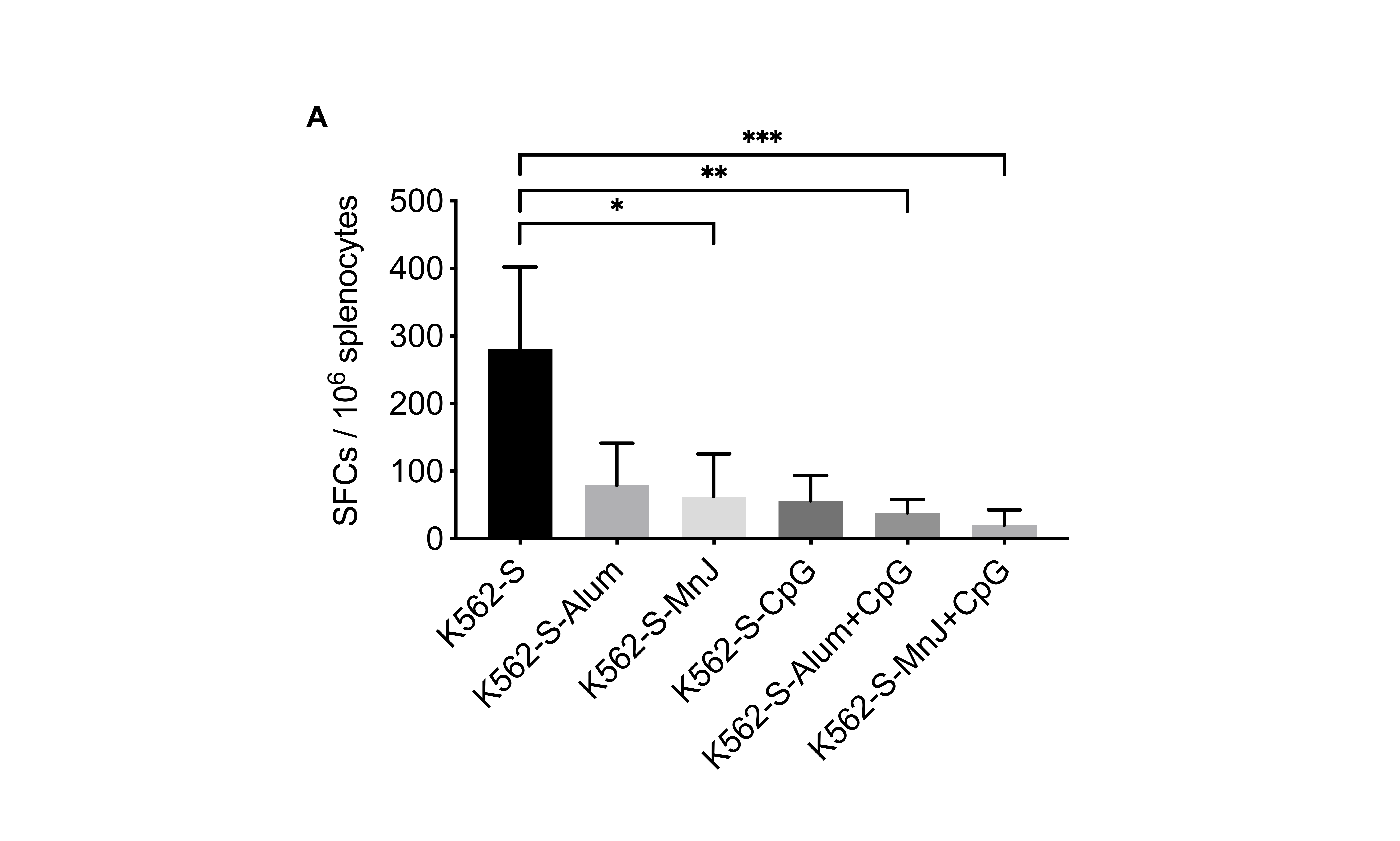

Supplement: Supplemental Material [file TEMI_A_1957400_SM4057.zip › Supplementary files/Fig.S4(LZW).tif]

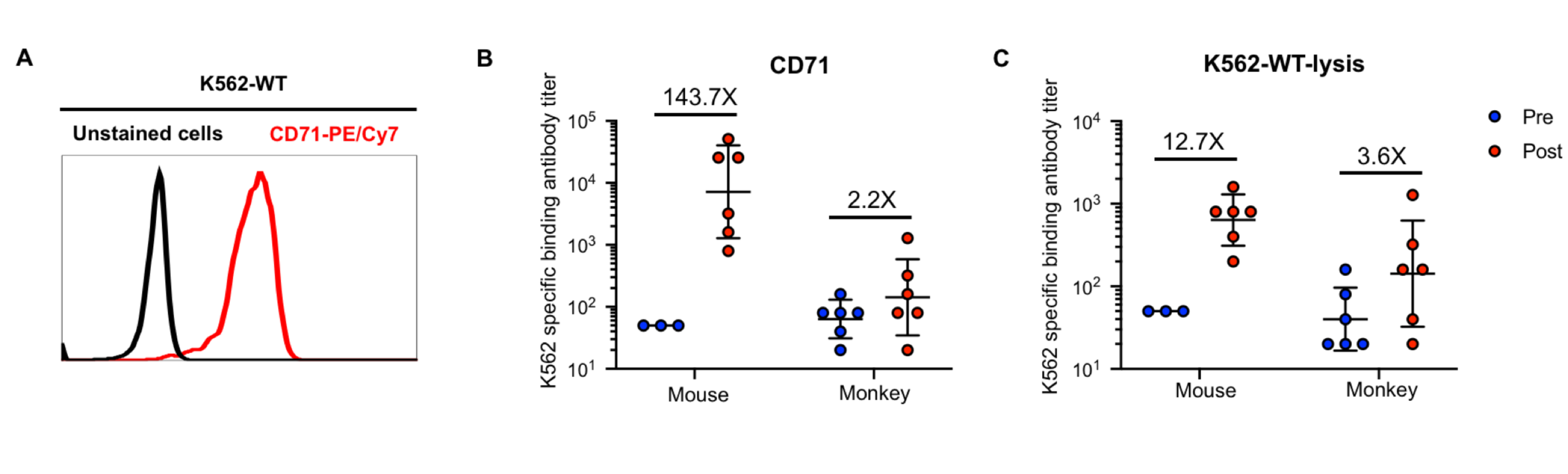

Supplement: Supplemental Material [file TEMI_A_1957400_SM4057.zip › Supplementary files/Fig.S6(LZW).tif]
